# Seasonal variations of leaf and canopy properties tracked by ground-based NDVI imagery in a temperate forest

**DOI:** 10.1038/s41598-017-01260-y

**Published:** 2017-04-28

**Authors:** Hualei Yang, Xi Yang, Mary Heskel, Shucun Sun, Jianwu Tang

**Affiliations:** 1 School of Life Sciences, Nanjing University, Jiangsu, 210093 China; 2 The Ecosystems Center, Marine Biological Laboratory, Woods Hole, Massachusetts 02543 USA; 3 Department of Earth, Environmental and Planetary Sciences, Brown University, Providence, RI 02912 USA; 4 Department of Environmental Sciences, University of Virginia, Charlottesville, VA 22904 USA

## Abstract

Changes in plant phenology affect the carbon flux of terrestrial forest ecosystems due to the link between the growing season length and vegetation productivity. Digital camera imagery, which can be acquired frequently, has been used to monitor seasonal and annual changes in forest canopy phenology and track critical phenological events. However, quantitative assessment of the structural and biochemical controls of the phenological patterns in camera images has rarely been done. In this study, we used an NDVI (Normalized Difference Vegetation Index) camera to monitor daily variations of vegetation reflectance at visible and near-infrared (NIR) bands with high spatial and temporal resolutions, and found that the infrared camera based NDVI (camera-NDVI) agreed well with the leaf expansion process that was measured by independent manual observations at Harvard Forest, Massachusetts, USA. We also measured the seasonality of canopy structural (leaf area index, LAI) and biochemical properties (leaf chlorophyll and nitrogen content). We found significant linear relationships between camera-NDVI and leaf chlorophyll concentration, and between camera-NDVI and leaf nitrogen content, though weaker relationships between camera-NDVI and LAI. Therefore, we recommend ground-based camera-NDVI as a powerful tool for long-term, near surface observations to monitor canopy development and to estimate leaf chlorophyll, nitrogen status, and LAI.

## Introduction

Plant phenology is the timing of key, annually occurring life history events, such as spring leaf development and autumn senescence, and the corresponding concurrent shifts in physiology. Understanding the response of phenology to global climate change, often observed in temperate forests as earlier spring and later autumn^1^, is crucial as the growing season length and annual vegetation-climate interactions affect ecosystem productivity, carbon fluxes, and other ecosystem functions. Therefore, long-term, high-resolution monitoring of plant phenology and seasonal variation in carbon fluxes across different scales is necessary for accurate estimation of the impact of environmental change on terrestrial carbon cycles^2^, especially in deciduous forest systems where vegetation is governed by seasonal patterns.

Recently, digital camera-based phenological monitoring (*e.g*. the PhenoCam network) has been adopted to capture seasonal canopy transitions and detect key phenological events at high spatial and temporal resolutions^1, 3–10^. Greenness indices can be calculated from digital camera images using composite red, green and blue pixel (together referred to as “RGB”) values, and applied to track the seasonal changes of canopy color^11^. However, visible-band digital cameras, which only use red, green and blue bands, may not accurately reflect certain plant physiological characteristics. Yang *et al*.^12^, Keenan *et al*.^13^, and Liu *et al*.^14^ found that the RGB camera-based phenological metrics were limited in their ability to characterize physiology: a critical decoupling existed between the peak of the camera-based greenness index and that of leaf chlorophyll concentration. Furthermore, RGB camera images can be strongly influenced by daily weather conditions, such as rain and fog^1, 15^.

Light absorption and reflection in leaves have been measured with many technological approaches—whether remotely from satellites or near-remotely from canopy towers—to estimate plant physiological processes. When photosynthesis occurs, green leaves absorb most of the incident visible radiation, particularly in the red band, and reflect and transmit most incident infrared light (NIR). Hence, a widely adopted vegetation index—Normalized Difference Vegetation Index (NDVI) based on red and infrared bands information—can be a robust metric for estimating photosynthetically active vegetation coverage, developmental status, and productivity^16^. Satellite-based NDVI (*i.e*. MODIS-NDVI) observes global vegetation growth and variation through growing seasons^17–19^. Hashemi and Chenani^20^ found that MODIS-NDVI produced a strong estimate of leaf chlorophyll concentration, and Zhang *et al*.^21^ proposed the use of remotely-sensed NDVI to monitor leaf nitrogen (N). However, to better understand climate-ecosystem interactions, satellite NDVI data need to be calibrated through ground-truthing to minimize error associated with variation in the community composition of plant species, shadows and background effects, and climatic variability.

To fully characterize the seasonal physiological variation of forest tree species and their impact on forest productivity, it is necessary to monitor and integrate plant processes at higher spatial and temporal resolutions. Within the last decade, hand-held NDVI cameras that can capture both visible and infrared wavelength bands were manufactured for research purposes, and many studies applied this technology to monitor crop yield and N status^22–24^. Petach *et al*.^25^ and Nijland *et al*.^26^ used NDVI cameras to monitor the plant phenological status, but linking the greenness index (infrared camera based NDVI, i.e. camera-NDVI) with leaf physiological traits has not been reported. Compared to satellite imagery, the NDVI camera offers freedom from cloudy conditions with high spatial and temporal resolutions. More recently, a simplified version of the NDVI camera using the light emitting diode (LED)-sensor technology has been reported to measure ground-based NDVI without reporting spatial information^27, 28^.

In this study, we monitored canopy phenology using an NDVI camera while collecting concurrent data on leaf physiological and chemical properties across the growing season. The major objective of this study is to explore how camera-NDVI correlates with leaf and canopy properties as well as with the manual phenological observations across the growing season in a deciduous forest.

## Results

### The seasonal variation of vegetation index

**Figure 1:**
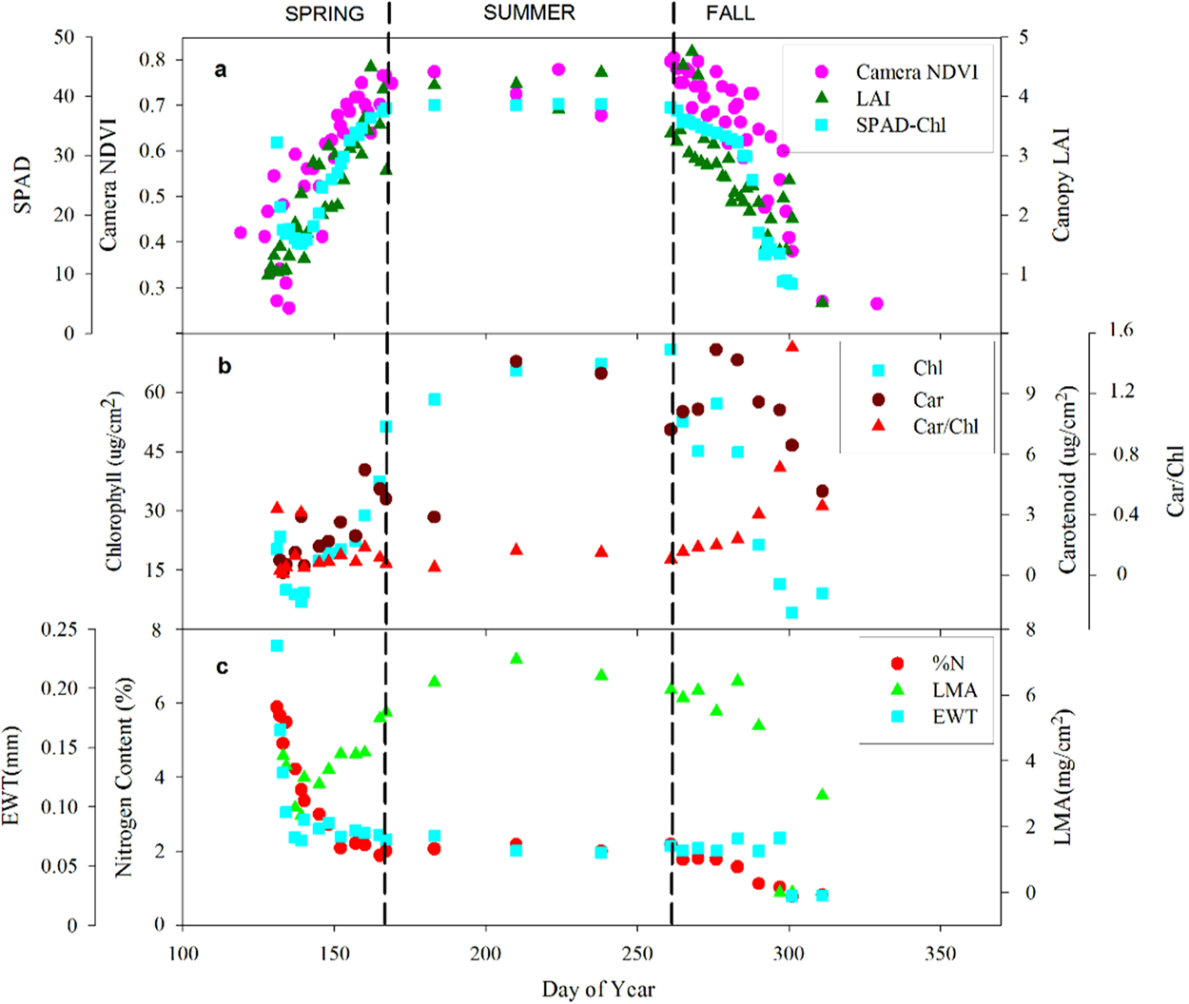
Seasonal changes of phenological metrics and leaf physiological properties. (**a**) Camera-NDVI, leaf area index (LAI) measured by LAI-2000 and leaf chlorophyll concentration measured by SPAD; (**b**) leaf total chlorophyll and carotenoids concentration, and their ratio (Car/Chl); (**c**) Leaf nitrogen content (%N), leaf mass per area (LMA) and Equivalent Water Thickness (EWT). The black dashed lines are the season dividers according to the seasonality of camera-NDVI.

**Figure 2:**
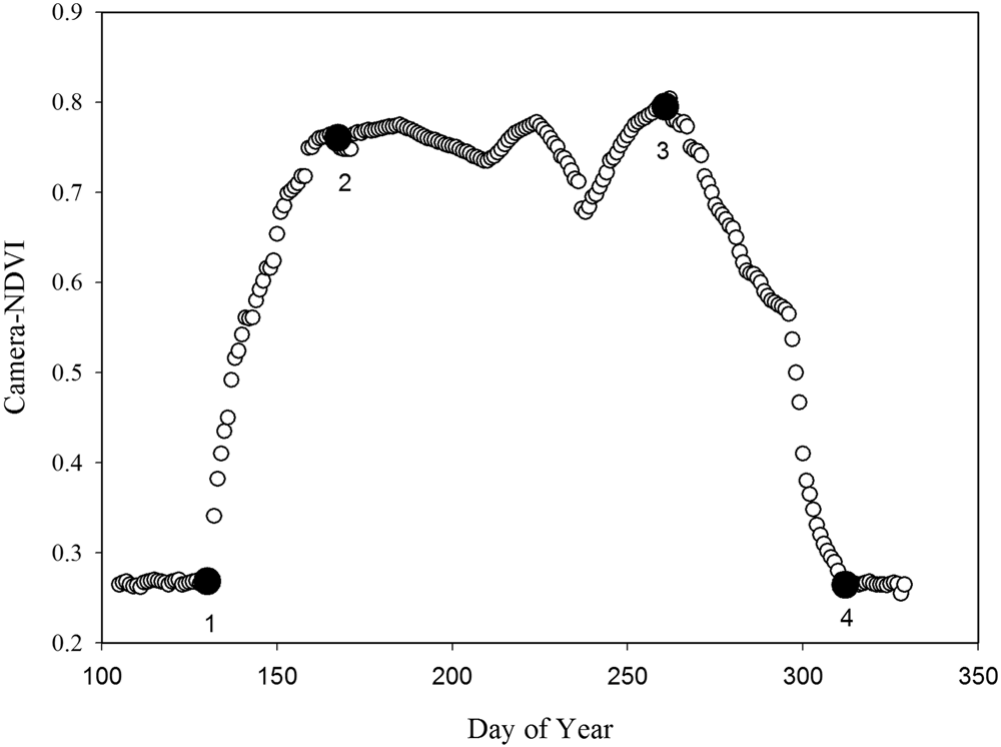
The seasonality parameters generated in TIMESAT from the smoothed time series of camera-NDVI: point 1 is beginning of season, point 4 is end of season, point 2 and 3 are the maximum values.

There was a symmetrical seasonal change of camera-NDVI values (Fig. 1a), reflecting an increase with the progression of spring leaf development and later, a decrease with autumn senescence of the canopy. Using TIMESAT-identified seasonal transition points (Fig. 2), we identified the date (approximately on DOY 132, or May 12^th^) of leaf-out when camera-NDVI abruptly increased after bud break, indicating the onset of rapid leaf expansion. Camera-NDVI peaked around DOY 167 (June 16^th^) in the early summer. Due to autumn coloration changes associated with senescence as well as leaf abscission, starting around DOY 262 (September 19^th^), values of camera-NDVI gradually declined and reached the lowest recorded values on DOY 311 (November 7^th^), when all leaves had fallen from the deciduous trees, and were completely absent in the ROI.

Our data showed that camera-NDVI tracked the leaf-out date (DOY 132), the canopy-greenness peak on DOY 167, and the leaf-off date (DOY 311), which agreed well with the seasonal trajectories of plant physiological and functional traits (see section 2.2).

### The seasonal variation of leaf and canopy properties

Camera-NDVI, the structural (LAI) and functional (chlorophyll and N content) properties of the canopy had similar seasonal changes during the growing season, except that camera-NDVI, LAI and SPAD-Chl increased, while %N decreased in the spring (Fig. 1).

At the beginning of the spring season, both SPAD-Chl (SPAD readings *in vivo*) and SPEC-Chl (extracted from leaf samples and subsequent spectrophotometric measurements) showed a small decline in chlorophyll concentrations after bud-break, then an increase during the whole spring season, and declined again from DOY 261. The only difference between SPAD-Chl and SPEC-Chl is that SPAD-Chl appeared more stable (fluctuation range: 37.5–38.5), while SPEC-Chl values increased slightly during the summer (Fig. 1a,b).

Overall leaf %N (mass-based) gradually declined at different developmental stages. Leaf %N fell from 5.6% at the beginning of spring to 0.8% in early winter, and was not reduced during the summer. EWT showed a similar seasonal decrease from 0.24 mm in early spring to 0.025 mm in early winter. LMA increased dramatically through the spring and summer seasons, reached to the peak 7 mg/cm^2^ in mid-summer, and then declined generally in autumn (Fig. 1c).

**Figure 3:**
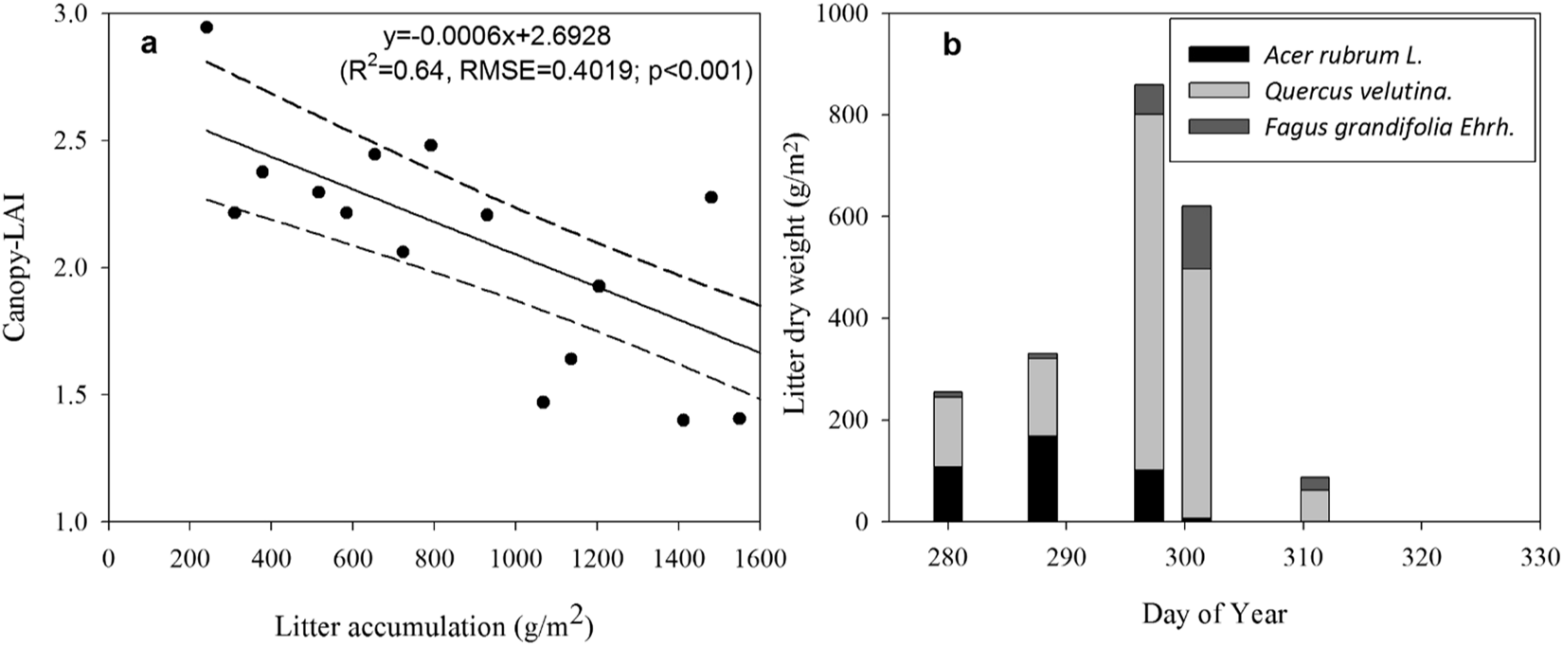
The process of autumn abscission. (**a**) The correlation between litter accumulation and canopy-LAI during leaf abscission (R^2^ = 0.64; p < 0.001), and the dotted lines represent 95% confidence interval; (**b**) Collected litter dry weights on October 7^th^, 15^th^, 24^th^, 28^th^ and November 7^th^, respectively.

Canopy-LAI showed similar pronounced seasonal variability (Fig. 1a). An increase of canopy-LAI occurred as more leaves emerged in the spring, and peaked around ~4.4 during summer, suggesting that the leaves completed expansion, and the canopy entered summer maturity on DOY 166. Around DOY 261, canopy-LAI began its decline associated with autumn senescence till a dormancy level. In autumn, canopy LAI decreased with the increasing of litter accumulation (R^2^ = 0.64; p < 0.001, Fig. 3a), reflecting the trajectories of leaf abscission, which mainly occurred in mid-to-late October (from October 15^th^ to 28^th^) in this region (Fig. 3b).

### Correlations between vegetation indices and plant physiological and functional traits

**Figure 4:**
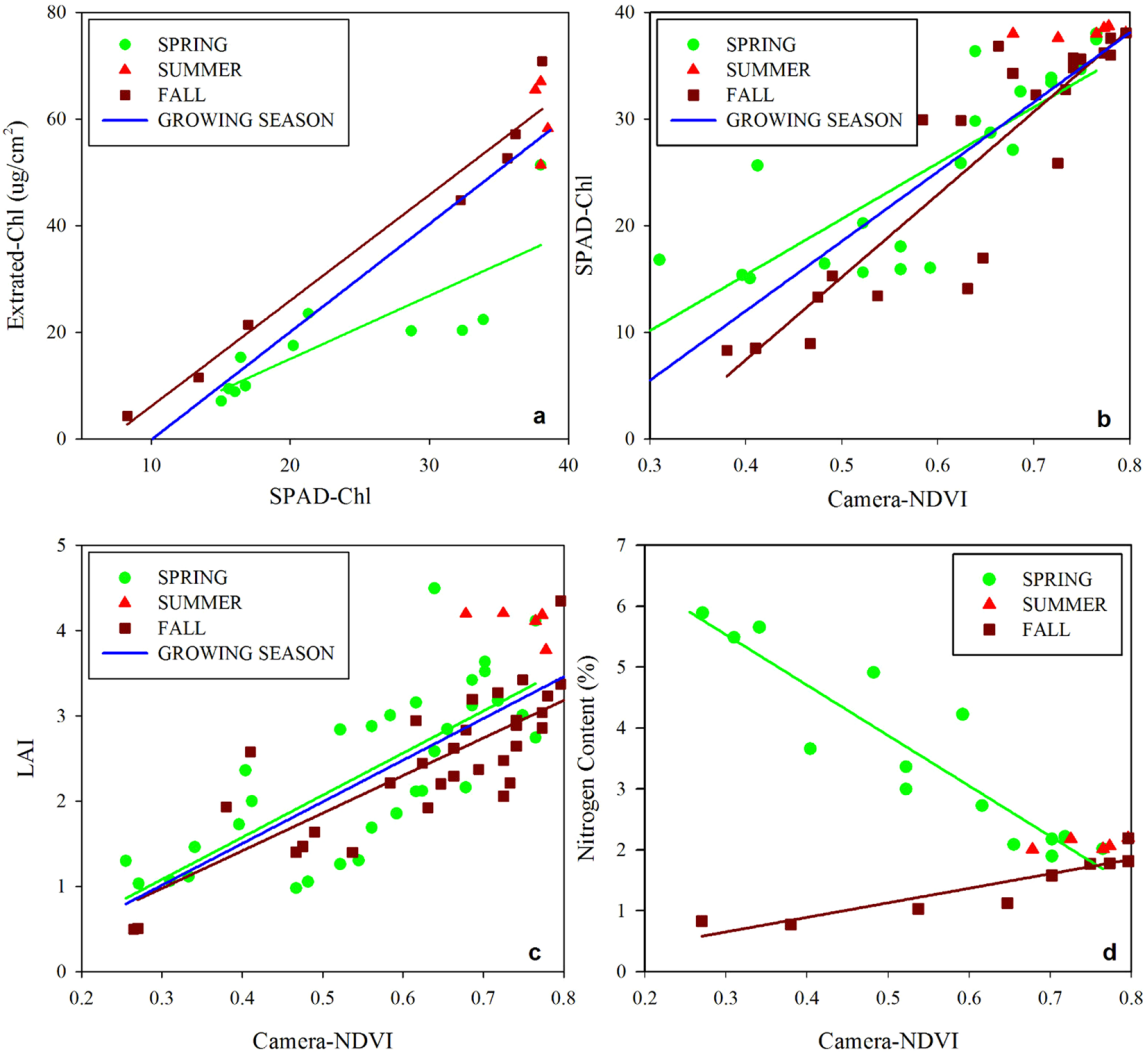
Linear regressions between camera-NDVI and plant physiological properties in different growing seasons. (**a**) SPAD-Chl and lab extracted Chl; (**b**) Camera-NDVI and SPAD-Chl; (**c**) Camera-NDVI and canopy LAI; (**d**) Camera-NDVI and leaf nitrogen content (%N) (See Table 1 for R^2^).

We quantified the linear relationships between SPAD-Chl and SPEC-Chl during different growing seasons: spring (R^2^ = 0.68), autumn (R^2^ = 0.96), and the entire growing season, including spring, summer, and autumn (R^2^ = 0.84, all p < 0.001) (Fig. 4a), demonstrating the reliability of SPAD to estimate the leaf chlorophyll concentration. Strong correlations were found between camera-NDVI and chlorophyll concentration (R^2^ = 0.67 in spring and R^2^ = 0.81 in fall; p < 0.001; Fig. 4b); overall, the slopes of this relationship were similar for spring and fall, as well as the entire growing season (Table 1).

**Table 1:** Linear regressions equations between camera -NDVI and plant physiological properties.

	Season	Equation	R^2^	p	RMSE	n
SPAD-Chl vs. Camera-NDVI	Spring	SPAD-Chl = 52.4022*NDVI − 5.573	0.67	< 0.001	5.2056	21
	Fall	SPAD-Chl = 77.4942*NDVI − 23.5825	0.81	< 0.001	4.7473	24
	Whole	SPAD-Chl = 65.2325*NDVI − 14.0824	0.73	< 0.001	4.6564	50
LAI vs. Camera-NDVI	Spring	LAI = 4.7887*NDVI − 0.3219	0.63	< 0.001	0.5385	32
	Fall	LAI = 4.4090*NDVI − 0.3440	0.61	< 0.001	0.5354	33
	Whole	LAI = 4.721*NDVI − 0.3286	0.62	< 0.001	0.5554	69
%N vs. Camera-NDVI	Spring	%N = −8.7392*NDVI + 8.3504	0.89	< 0.001	0.4975	14
	Fall	%N = 2.3845*NDVI − 0.0603	0.82	< 0.001	0.1995	9

Canopy LAI showed a close correlation with camera-NDVI in the spring and fall (Fig. 4c). A general linear trend was observed between camera-NDVI and LAI (R^2^ = 0.63 in spring and R^2^ = 0.61 in fall; p < 0.001), and slopes were equivalent in spring, fall and the entire growing season (Table 1). Camera-NDVI was saturated during the summer season where canopy-LAI was greater than 4.

Strong linear relationships were also found between camera-NDVI and %N (R^2^ = 0.89 in spring and R^2^ = 0.82 in fall; p < 0.001) (Fig. 4d). A seasonal decoupling existed between camera-NDVI and %N throughout the growing season, showing a negative correlation in spring and a positive correlation in autumn. The absolute value of the slope of the linear regression in spring was much steeper than that in fall (Table 1).

### Correlations between camera-NDVI and manual phenological observations

**Figure 5:**
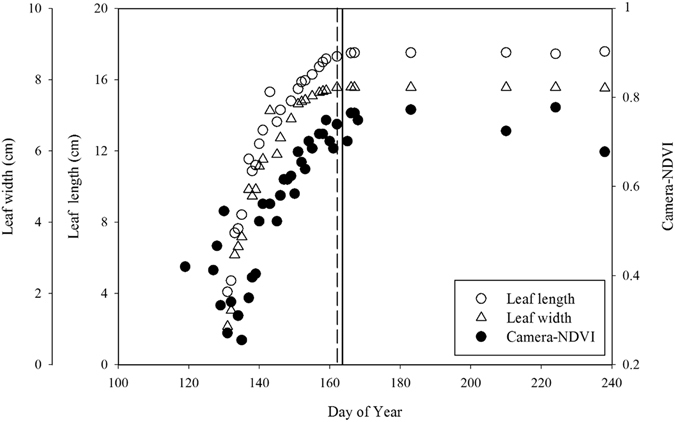
The process of leaf expansion in spring green-up. The solid line and dash line stand for the change point probability (CPP) extracted by the Bayesian multiple change point analysis of camera-NDVI and leaf size (length and width) in late spring, respectively.

In the spring green-up season, leaf length and width increased with leaf emergence and expansion, and their seasonal change point probability (CPP) appeared both on DOY 161 ± 1. Meanwhile, the CPP of camera-based NDVI (DOY 162 ± 1) in the late spring was close to that of leaf length and width (Fig. 5).

## Discussion

This study used a ground-based imagery approach (an infrared NDVI camera) to continuously investigate the seasonality of canopy development and physiological traits (chlorophyll concentration, leaf N status and LAI) throughout the growing season in a temperate deciduous forest.

Canopy-NDVI showed seasonal patterns (Fig. 1) that indicated spring green-up and autumn senescence processes in a deciduous forest. According to the increasing and decreasing trends of camera-NDVI, we identified DOY 132–167 as spring, DOY 168–261 as summer, and DOY 262–311 as the autumn season in 2014 (Fig. 2). These patterns match well with the data of leaf traits. The abnormal fluctuations of camera-NDVI between DOY168 and DOY 261 in summer (Fig. 2) may be due to that there were only 4 NDVI values during the summer (Fig. 1a), not necessary in representing the real dynamic of camera-NDVI. Moreover, this camera-based NDVI imagery method allows researchers to select the special “region of interest,” which can be used to answer questions about species diversity and spatial heterogeneity, while the tower-based LEDs-sensor can only measures an aggregated NDVI for a region. Another important index based on the infrared and red bands is the enhanced vegetation index (EVI) that has been proposed to replace NDVI to avoid the greenness saturation issue^29^. In this study, we calculated EVI2 (without the blue band) but found that there was no significant difference from NDVI.

During the spring green-up, with the leaf expansion (i.e. leaf length and width increased), values of camera-NDVI increased at a rapid rate (Fig. 5). The seasonal CPP of camera-based NDVI (DOY 162 ± 1) in the late spring was similar to that of leaf length and width (leaf length had a proportional increasing to leaf width in this study) (Fig. 5), suggesting camera-NDVI could accurately detect the spring leaf expansion process.

Canopy LAI, leaf chlorophyll, and N concentration are key structural and functional parameters that link directly to photosynthetic potential and primary production of forest canopies^15, 30, 31^. These variables can also provide indications of the physiological status or stress^32, 33^. Thus, developing a more accurate and effective approach to continuously investigate the seasonality of these physiological properties can be useful for future applications.

In this study, a significant relationship was found between camera-NDVI and leaf chlorophyll concentration throughout leaf development (Fig. 4b), suggesting that the infrared band can be used to estimate the dynamic of leaf chlorophyll status. In the spring, camera-NDVI matched with the increasing of chlorophyll (Fig. 4b). During senescence, leaf chlorophyll production and photosynthesis cease, and therefore measures of chlorophyll using both methods (SPAD-Chl and SPEC-Chl) synchronously decreased (Fig. 1a,b). Due to the faster disassembly of chlorophyll compared to carotenoid in leaves, the ratio of carotenoid and chlorophyll concentrations (Car/Chl) - which can indicate the functional pigment capacity and the greenness of plants^34^ – increased and corresponded to the reddening of canopy leaves that lead to the decline in camera-NDVI values (Fig. 2). Higher values of camera-NDVI in autumn compared to values in spring may be explained by the corresponding higher leaf chlorophyll concentration in autumn (Fig. 4b). Variations in the total chlorophyll or carotenoids can also indicate environmental stress in plants^35^, as well as senescence and damage. Chlorophyll readings made with SPAD can be used to monitor dynamic changes, since the SPAD values are highly correlated with lab extracted leaf chlorophyll concentration^35–38^ (Fig. 4a). We found an inconsistency between SPAD-Chl and extracted-Chl in the timing and duration of peak values; SPAD-Chl maintained the peak value throughout the summer season, while extracted-Chl reached the maximum in mid-summer (Fig. 1a,b). This may indicate that the SPAD reading is less sensitive at higher chlorophyll concentrations^39^ due to high heterogeneity in chlorophyll distribution within the leaf^40^. Naus *et al*.^39^ and Ling *et al*.^41^ reported that the SPAD-Chl differs from solvent-extracted chlorophyll by ~6%. Monje and Bugbee^42^ found that SPAD-Chl showed sensitivity to chlorophyll concentrations in leaves with extracted-Chl below a certain level, and above that level, the sensitivity of the SPAD-Chl to extracted-Chl was considerably reduced.

Nitrogen is a major component of chlorophyll and associated photosynthetic enzymes in plants, and provides essential nutrients for growth^43–45^. Significant correlations were observed between camera-NDVI values and leaf N concentration, and thus visible and near infrared spectral reflectance (NDVI) values may be able to provide important information on leaf biochemical properties throughout the growing season^46^. The relationship between camera-NDVI and leaf N supports the potential for remote estimation of leaf N^47–49^. The seasonal changes of leaf N concentration are closely related to plant growth stages and physiological activity^45^. At the onset of the growing season, leaf N concentration peaked after leaf-out, and then declined to a stable status in the mid-season during a period of biomass accumulation (Fig. 1c). In fall, the lower slope of the relationship between leaf N and camera-NDVI indicates that leaf N decreased at a slower rate during senescence and abscission in deciduous species. During senescence, a fraction of leaf N is transferred to other plant tissues and stored for the following spring leaf-out^50^. Seasonal decreases in leaf N concentration are likely due to a dilution effect associated with increases in LMA and decreases in EWT (Fig. 1c). It should be noted that when using camera-NDVI to estimate mass-based leaf N, it is necessary to account for seasonal variability, due to the seasonal difference in the relationship between camera-NDVI and %N in spring, summer, and autumn (Table 1; Fig. 4d). The season-specific relationships between camera-NDVI and leaf N can complicate estimations that work under an assumption of a constant relationship obtained from peak-growing season data, further emphasizing the importance of integrating shoulder-season observations of forest function.

The parallel seasonal trajectory between camera-NDVI and canopy leaf area index (LAI) demonstrated an application of an NDVI camera for investigating canopy LAI at high spatial resolution (Fig. 4c). The linear relationships between camera-NDVI and LAI were similar for both spring and fall seasons (Table 1). We also found that camera-NDVI became saturated at high LAI values during the summer. This indicated that the NDVI-camera had the potential to reflect the seasonal and inter-annual variability of vegetation coverage and canopy development that are closely related to canopy photosynthetic capacity and gross primary productivity in a terrestrial ecosystem^22–24, 51^, as has been shown in other deciduous broad-leaved forests^9, 37, 52^.

Camera-NDVI values are closely correlated with leaf chlorophyll and N content and LAI across the growing season (Table 1), which may be attributed to the absorption of most incident visible radiation by green leaf material, particularly in the red band, and the subsequent reflection and transmission of most incident NIR. Ecosystem structural and functional properties show specific responses to visible and infrared reflectance^28, 51, 53^. Claude and Bellefleur^54^ and Card *et al*.^55^ observed that leaf N concentration was more closely correlated with reflectance in the red than in the green region of the plant spectrum. Previous studies also used the vegetation indices (such as NDVI and EVI) to estimate LAI and compare with leaf chlorophyll and nitrogen contents^29, 56–58^, and found positive correlations between the vegetation indices and LAI and leaf chlorophyll content, consistent with our results. It is noteworthy that they found a positive relationship between the vegetation indices and the leaf area-based N content, while we reported the negative relationship between camera-NDVI and the mass-based N content, suggesting a dramatic increase in leaf mass per area (*LMA*) during the spring green-up (Fig. 1c) that causes an decrease in mass-based N content but an increase in area-based N, a consistent pattern between this study and previous studies^29, 56, 58^.

This study did not directly scale leaf-level NDVI to canopy-level NDVI. To do so, canopy radiative transfer models (such as the PROSPECT and SAIL models) must be used to simulate leaf- and canopy-level reflectance spectral curves^59^. Due to the advantage of NDVI-cameras in monitoring plant phenology and biophysical parameters, adding the NDVI cameras to the phenology network in addition to RGB cameras (i.e. adding NIR band into regular RGB cameras) may improve the accuracy of vegetation phenological observation. However, due to the higher price of NDVI cameras compared with traditional RGB cameras, this technique may be difficult to be widely used. Another economical way as an alternative to NDVI cameras is to add the infrared bands into regular RGB cameras through specialized infrared filters that allow infrared light to pass through while blocking the visible light. For instance, the raw RGB image could be converted into a red, green and NIR false-color image with a blue-blocking filter^22^.

In summary, the high spatial and temporal resolutions of NDVI cameras that combine visible and NIR bands can provide continuous information on canopy phenology and physiology and inform ecosystem canopy models about the timing of physiological processes during the growing season. The infrared signals can indicate not only the expected phenological pattern but also subtle variables of canopy structure (LAI) and leaf functional traits (leaf chlorophyll concentration and %N) during the growing season. We found that the NDVI camera showed the high correlations with leaf chlorophyll, N status, and LAI. As the reflectance in the visible and near-infrared wavelengths is correlated with terrestrial vegetation status and growth, linking camera-NDVI with vegetation structure and function can advance the understanding of ecosystem processes and biosphere-atmosphere interactions^60^. We concluded that camera-NDVI was useful to identify the key phenological events, such as leaf-out, leaf expansion process, greenness peak and leaf-off dates. The infrared camera provided a new tool with a wide range of wavebands to interpret the seasonality of vegetation structural and functional properties.

## Methods

### Site description

The study was conducted at the Harvard Forest, Petersham, Massachusetts, USA (42°32′6″N, 72°10′28″W) during the 2014 growing season (~May-October). This forest site is characterized by a cool, temperate climate with average temperatures of −7 °C in January and 23 °C in July; average annual precipitation is ~110 cm, distributed evenly throughout the year. The study site is located in an approximately 80–100 years old mixed hardwood stand dominated by red oak (*Quercus rubra*), red maple (*Acer rubrum L*.), and American beech (*Fagus grandifolia Ehrh*.). Snow typically covers the ground for several months during winter. Soils are mainly sandy loam glacial till, with some alluvial and colluvial deposits. All camera imagery was collected above the canopy from the top platform of the hardwood walk-up tower (around 28 m or approximately 5 m above the forest canopy).

### NDVI camera-based canopy phenological observations

**Figure 6:**
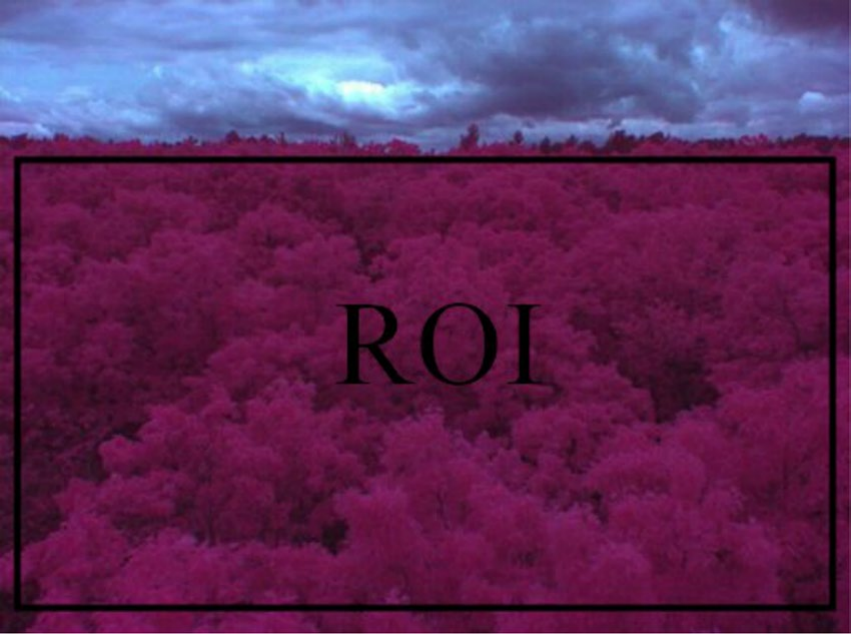
The example of an infrared image on September 19^th^, 2014. The black rectangles indicate the Region of Interest (ROI) used to calculate camera-NDVI. The ROI includes approximately 2,800,000 pixels. Because plants absorb much red light and reflect a lot of infrared light, this NDVI camera makes near infrared wavelengths visible as red while red wavelengths appear as green and green as blue, and thus produced a new color-infrared (CIR) image. On CIR imagery vegetation appears red.

NDVI images (Fig. 6) were manually taken with a Tetracam Agriculture Digital Camera (Tetracam, Inc., Chatsworth, CA. (www.tetracam.com)), which contains a single precision 3.2 megapixel image sensor (storing 2,048 × 1,536 pixels), to characterize changes in canopy greenness during the growing season. The camera can also be set as an automated mode to take images. The footprint of the captured image was approximately 150 × 200 m in length and width at the ground level. A blue absorbing glass filter was used to eliminate the blue sensitivity, and the blue pixels in the sensor were used to measure near-infrared (NIR) reflectance. A color-infrared (CIR) image with NIR, red, and green bands was produced from this imaging. The infrared images were processed by software PixelWrench2 (Tetracam, Inc., Chatsworth, CA, USA) to calculate the NDVI values in the selected region of interest (ROI), which covered the whole canopy (Fig. 6). To calibrate values representing canopy color, images of a Teflon calibration tag were photographed under the same lighting conditions as the canopy images in order to reduce the influence of different light environments. NDVI images of the forest canopy were shot at midday every day from the top platform of the hardwood walk-up tower (viewing zenith angle: 30°) in spring (May 7^th^–June 18^th^) and autumn (September 18^th^–October 30^th^) seasons and twice a month during the summer. The ROI was adjusted if needed to ensure images covering the same location over time were processed. Normalized Difference Vegetation Index (NDVI) is defined as follows:

1 $${\rm{NDVI}}=({\rm{NIR}}-{\rm{RED}})/({\rm{NIR}}+{\rm{RED}})$$

where RED and NIR stand for the spectral reflectance in the visible red and near-infrared regions, respectively.

We used TIMESAT (version 3.2, http://web.nateko.lu.se/timesat/timesat.asp), a software package processing remotely sensed time series, to analyze the time series of the NDVI data and obtain the seasonality information of vegetation. The NDVI time series were smoothed using the Savitzky-Golay filter^61^. Then we applied TIMESAT to extract the beginning of season (Point 1), end of season (Point 4), the length of season (the distance between point 1 and 4), and the maximum value (point 2 and 3) from the smoothed time series (Fig. 2).

### Leaf chlorophyll concentration

We used two approaches to monitor changes of chlorophyll concentration during the growing season: non-destructive and destructive. For non-destructive chlorophyll estimation, we used a Soil Plant Analysis Development (SPAD)-502 meter (Spectrum Technologies, Aurora, IL, USA), which is widely used as a rapid and accurate tool that utilizes leaf transmittance in two wavebands centered at 650 nm and 940 nm^36^. We selected ~15 leaves from top part of canopy (16–23 m) of the *Quercus rubra*, *Acer rubrum L*. and *Fagus grandifolia Ehrh*. For each leaf, made five SPAD readings that were evenly distributed over the whole leaf area and then averaged for each leaf. Leaves were sampled daily during the shoulder seasons, and biweekly during the peak growing season.

The destructive method to measure the chlorophyll concentrations based on the absorption of light by 90% Acetone solution and MgCO_3_ mixture at laboratory^62^. Throughout the growing season (weekly in spring and autumn, and biweekly in summer), about 12 leaf samples (of *Quercus rubra*, *Acer rubrum L*. and *Fagus grandifolia Ehrh*.) were sampled from the upper canopy (16–23 m) and then several leaf discs were punched from each leaf using a hole puncher (~0.2827 cm^2^ each). Three leaf discs were pestled in a mortar to mix samples^12, 53^. Before run in a spectrophotometer, the samples were centrifuged for 8 minutes on a high level to isolate the supernatant and sediment. The absorbance of the supernatant at 470, 645 and 662 nm wavelengths^63^, respectively, were measured using the spectrophotometer (Shimazu UV-1201, Kyoto, Japan). Leaf pigment concentrations, specifically chlorophyll a, chlorophyll b, and total carotenoid, in the extract solution were calculated^63^.

### Leaf area index (LAI)

During the growing season (daily in spring and autumn, and biweekly in summer; twice per day and averaged for daily LAI), leaf area index (LAI) of the forest was measured using an LAI-2000 Plant Canopy Analyzer (LI-COR, Inc., Lincoln, NE, USA). All measurements were made when the sun was near the horizon (before sunrise or after sunset), or on overcast days to reduce the contribution of scattered radiation.

To track leaf abscission in autumn, ten litter traps (50 × 50 × 35 cm) were arranged on the forest floor within the canopy tower footprint to collect abscised leaves. We collected leaf litter from these traps, which included leaves of *Acer rubrum L*., *Quercus rubra* and *Fagus grandifolia Ehrh*, on October 7^th^, 15^th^, 24^th^, 28^th^ and November 7^th^, and weighed litter dry mass after oven-drying at 70 °C for 48 hours.

### Leaf nitrogen content, equivalent water thickness, and leaf mass per area

After budbreak, leaf samples of *Acer rubrum L*., *Quercus rubra* and *Fagus grandifolia Ehrh*. were collected for chlorophyll concentration analysis, additional leaves (*n* = 10–12) of the top canopy were sampled concurrently to assess leaf water status and N content. After cutting, fresh leaves were weighed immediately (*M*_F_) and scanned with a digital scanner (EPSON V300, EPSON, Long beach, CA, USA), and area (*LA*) was calculated using ImageJ software. Subsequently, leaves were oven-dried at 70 °C for 48 hours prior to measuring leaf dry mass. After weighing dry mass (*M*_D_), the dry leaf samples were used to determine leaf nitrogen content (N mass percentage for the leaf sample, %N) by the Thermo Scientific CN Analyzer (FLASH 2000, Thermo scientific, Waltham, MA, USA). Leaf mass per area (*LMA*) and equivalent water thickness (*EWT*)^12^ were calculated as:

2 $$LMA={M}_{D}/LA$$

3 $${\rm{EWT}}=({M}_{F}-{M}_{D})/({{\rm{d}}}_{{\rm{w}}}\ast LA)$$

where d_w_ is the density of water (1 g/cm^3^).

### Manual phenological observations

During spring green-up, we measured the length and width of leaves to track the process of leaf expansion. Daily mean leaf length and width of 15 top canopy leaves of the study species (*Quercus rubra* and *Fagus grandifolia Ehrh*.) were averaged. We detected the change point probability (CPP) of the spectral vegetation index (camera-based NDVI) and leaf dimensions (leaf length and width) during spring expansion using the Bayesian multiple change point analysis, which was applied to detect major transition dates indicating when the phenological events occur within a climate time series^14, 64^.
